# Tian-Huang Formula, a Traditional Chinese Medicinal Prescription, Improves Hepatosteatosis and Glucose Intolerance Targeting AKT-SREBP Nexus in Diet-Induced Obese Rats

**DOI:** 10.1155/2021/6617586

**Published:** 2021-03-06

**Authors:** Kun-Ping Li, Yang Yu, Min Yuan, Chu-Mei Zhang, Xiang-Lu Rong, Jeremy E. Turnbull, Jiao Guo

**Affiliations:** ^1^Guangdong Metabolic Diseases Research Center of Integrated Chinese and Western Medicine, Guangdong Pharmaceutical University, Guangdong TCM Key Laboratory for Metabolic Diseases, Guangzhou 510006, China; ^2^Guangdong International Cooperation Base for Prevention and Treatment of Metabolic Diseases, Guangzhou 510006, China; ^3^Key Laboratory of Glycolipid Metabolic Diseases, Ministry of Education, Guangzhou 510006, China; ^4^Department of Biochemistry, Institute of Integrative Biology, University of Liverpool, Liverpool L69 7ZB, UK

## Abstract

The progressive increase of metabolic diseases underscores the necessity for developing effective therapies. Although we found Tian-Huang formula (THF) could alleviate metabolic disorders, the underlying mechanism remains to be fully understood. In the present study, firstly, male Sprague-Dawley rats were fed with high-fat diet plus high-fructose drink (HFF, the diet is about 60% of calories from fat and the drink is 12.5% fructose solution) for 14 weeks to induce hepatosteatosis and glucose intolerance and then treated with THF (200 mg/kg) for 4 weeks. Then, metabolomics analysis was performed with rat liver samples and following the clues illustrated by Ingenuity Pathway Analysis (IPA) with the metabolomics discoveries, RT-qPCR and Western blotting were carried out to validate the putative pathways. Our results showed that THF treatment reduced the body weight from 735.1 ± 81.29 to 616.3 ± 52.81 g and plasma triglyceride from 1.5 ± 0.42 to 0.88 ± 0.33 mmol/L; meanwhile, histological examinations of hepatic tissue and epididymis adipose tissue showed obvious alleviation. Compared with the HFF group, the fasting serum insulin and blood glucose level of the THF group were improved from 20.77 ± 6.58 to 9.65 ± 5.48 mIU/L and from 8.96 ± 0.56 to 7.66 ± 1.25 mmol/L, respectively, so did the serum aspartate aminotransferase, insulin resistance index, and oral glucose tolerance (*p* = 0.0019, 0.0053, and 0.0066, respectively). Furthermore, based on a list of 32 key differential endogenous metabolites, the molecular networks generated by IPA suggested that THF alleviated glucose intolerance and hepatosteatosis by activating phosphatidylinositol-3 kinase (PI3K) and low-density lipoprotein receptor (LDL-R) involved pathways. RT-qPCR and Western blotting results confirmed that THF alleviated hepatic steatosis and glucose intolerance partly through protein kinase B- (AKT-) sterol regulatory element-binding protein (SREBP) nexus. Our findings shed light on molecular mechanisms of THF on alleviating metabolic diseases and provided further evidence for developing its therapeutic potential.

## 1. Background

Obesity and its close consequences, dyslipidemia, impaired glucose tolerance, and insulin resistance, are more common than ever in human history [1]. The progressive increase of the above conditions leads to the rising incidence of type 2 diabetes mellitus (T2DM), nonalcoholic fatty liver disease (NAFLD), and metabolic syndrome [2]. Overnutrition and lack of physical activities are usually regarded as the key elements of obesity, and a dietary pattern preferring a high-fat high-sugar diet plays a central role among the determinants of obesity in modern society [3, 4]. Epidemiological evidence showed a clear correlation between the increased consumption of a Western high-calorie diet and high-fructose corn syrup (HFCS) and the incidence of obesity and its complications [3–5]. A number of countermeasures have been applied to fighting against these situations, including active lifestyle modifications, pharmacotherapeutic intervention, and even aggressive bariatric surgery [2, 6]. Noninvasive drugs have been regarded as promising approaches to address the deficit of lifestyle intervention and reduce the severity of bariatric surgery [2, 6, 7]. Although varying in their efficacy and side effect profiles, the existing available approved drugs targeting obesity and its close consequences and medical comorbidities are inadequate. Meanwhile, the complexity of obesity and associated comorbidities underscore the necessity for developing suitable drugs with different benefits in the context of our understanding of the physiopathology of obesity [2, 6]. As is well known, the pharmacological properties of many traditional herbal medicines have been demonstrated according to hundreds of years' experience on humans. In addition, many kinds of natural plant extract derived from them have been developed and used as dietary supplements, though knowledge on their molecular mechanisms is lacking [8, 9]. Therefore, developing novel therapeutics from natural herbal extracts is a promising strategy to address the global health problem of obesity and its consequences; further studies on the precise role and mechanism of potential drugs against the development of diet-induced metabolic diseases will provide better support for their clinical usage [10].

Tian-Huang formula (THF) is a patented and clinically approved Chinese medicinal prescription with hypolipidemic effects. It originated from Traditional Chinese Medicine Fufang Zhenshu Tiaozhi Formula (FTZ) which was derived from Prof. Jiao Guo's 30 years of clinic experience and has been developed into hospital preparations. Due to its excellent cost-effective properties, FTZ capsules have been covered by health insurance in Guangdong Province, China. In the past few years of clinical and experimental study, a more simple but equivalent formula originated from FTZ, namely, THF, was developed [11]. It is composed of *Panax notoginseng* and *Coptis chinensis*, which are both traditional herbal drugs with hundreds of years of usage [12, 13]. Total saponins of *P*. *notoginseng* (PNS) and total alkaloids of *C*. *chinensis* (CCA) are believed to be the main active ingredients of *P*. *notoginseng* and *C*. *chinensis*, respectively [12, 13]. Gu et al. showed that the active constituents of PNS were ginsenoside Rb_1_, ginsenoside Rg_1_, ginsenoside Rb, ginsenoside Re, and ginsenoside R_1_ [14]. It has been demonstrated that PNS ameliorates hepatic lipid accumulation [15] and hepatic diseases [16]. Nowadays, preparations made from PNS are available and widely used in clinics in China [17]. Meanwhile, berberine, palmatine, and coptisine were reported to be the main active constituents of CCA [18], which has anti-hyperglycemic and anti-hyperlipidemic effects and can be used for anti-diabetic treatment [19, 20]. Our previous rodent model-based experiments showed that THF had therapeutic effects on lipid lowering and anti-atherosclerosis [11]. However, the potential mechanisms for improving glycolipid metabolism of THF have not been fully elucidated.

Metabolomics analysis is emerging as a robust tool for studies on diagnostic biomarkers, fundamental pathogenic mechanisms, and therapeutic targets [21–23] According to biochemical understanding, endogenous metabolites are products of all kinds of life-sustaining biochemical reactions, which can reflect alterations of the bodies' homeostasis. Moreover, use of software such as IPA (Ingenuity Pathways Analysis), MetaCore, and Reactome, algorithmically constructed metabolic networks can be generated to provide more insight than phenotypes and results analyzed individually. They can then provide clues to the pathophysiology of diseases and drug intervention research [24–26].

In the present study, in order to provide mechanistic insight into the therapeutic effects of THF on metabolic disorders, a nontargeted metabolomics analysis and IPA analysis have been performed. Subsequent results validated that THF alleviates glucose intolerance and hepatic steatosis by targeting AKT- (protein kinase B-) SREBP (sterol regulatory element-binding protein) nexus through activating PI3K (phosphatidylinositol-3 kinase) and LDL-R (low-density lipoprotein receptor) involved pathways. Our results shed new light on the underlying mechanisms and present informative evidence that AKT-SREBP nexus is a potential therapeutic target of THF for metabolic disturbances, and furtherly could offer new molecules to therapeutically intervene DIO and its complications.

## 2. Methods

### 2.1. Chemicals

High-fructose corn syrup (F55) was supplied by Guangzhou Shuangqiao Co., Ltd. (Guanzhou, China). Nonadecanoic acid was purchased from Sigma-Aldrich (Sigma, San Diego, USA). Methanol, hexane, and chloroform were of HPLC (High Performance Liquid Chromatography) grade and were from Merck (Darmstadt, German). Pyridine, O-methylhydroxylamine hydrochloride, N-methyl-N-(trimethylsilyl) trifluoroacetamide (MSTFA), and trimethylchlorosilane (TMCS) were from J&K Scientific Co. Ltd. (Tianjing, China).

### 2.2. Preparation and Quantitative Profiling of THF

THF was prepared as previously described [11]. In brief, powdered *P*. *notoginseng* (400 g) and *C*. *chinensis* (400 g) were separately extracted triply with 70% ethanol at 80°C under reflux, each time for two hours. The extract solution was concentrated in a rotary evaporator to remove ethanol and then dissolved in water and purified using D101 macro-porous resin (Lanxiao, Xi'an). The resulting purified extract was dried in vacuum at 60°C.

The quantitative profiling of THF was performed on an U3000 HPLC with a DAD detector (Dionex, USA). The chromatograph separation was carried out using a Kromasil C18 column (4.5 × 250 mm, 5 *μ*m in particle size) according to the Pharmacopoeia of People's Republic of China (Ch. P. 2015), and data were recorded and analyzed on Chromeleon Console workstation (for details, see the Supplementary materials). Finally, the contents of eight active components in THF, namely, ginsenoside Rg1, ginsenoside Rb1, ginsenoside Rd, ginsenoside Re, notoginsenoside R1, berberine, coptisine, and palmatine, were quantified.

### 2.3. Animals

Five-week-old male Sprague-Dawley (SD) rats were supplied by Guangdong Medical Laboratory Animal Centre (Guangzhou, China). Rats were housed in a plastic cage (4∼5/cage) and a specific pathogen-free facility at controlled temperature of 24 ± 2°C, with relative humidity of 60–70% and a 12-hour light-dark cycle. All animals had free access to water and standard rodent diet during acclimatization. All procedures performed were approved by the Experimental Animals Ethics Committee of Guangdong Pharmaceutical University (No. SPF2017092). All animal studies were conducted in accordance with the ARRIVE (Animal Research: Reporting of *In Vivo* Experiments) guidelines for reporting experiments involving animals [27].

### 2.4. Study Design

After one week of acclimatization, all rats were randomly assigned as control group (Cont, *n* = 8) and test group (*n* = 16). The test group were fed a high-fat diet ad libitum for 14 weeks. The test group had continuous access to a separated bottle with high fructose corn syrup solution (HFCS-55: 55% fructose, and 45% glucose, diluted with distilled water to 12.5% fructose solution). The standard diet provided 20% of calories from protein, 70% of calories from carbohydrate, and 10% of calories from fat, and a digestible energy of 3.85 kcal/g (D12450-B). The high-fat diet provided 20% of calories from protein, 20% of calories from carbohydrate, and 60% of calories from fat, and a digestible energy of 5.24 kcal/g (D12492). The body weight of rats was measured and food intake was recorded every week. During the study period, plasma triglyceride, total cholesterol (TC), low-density lipoprotein-cholesterol (LDL-C), and high-density lipoprotein-cholesterol (HDL-C) concentrations were determined at week 8, 12, and 14. At the end of week 14, the test group rats were divided into two groups, the HFF group and vehicle-treated group (HFF, *n* = 8), the HFF-fed and THF-treated group (200 mg/kg; THF, *n* = 8), according to the body weight, plasma TG (triglyceride), and TC levels.

After treating with THF for additional 4 weeks, all the rats were sacrificed after anesthetizing with pentobarbital sodium to collect the blood from the visual abdominal aorta after a 14-hour overnight fast. Blood was conducted with heparin and centrifuged at 4°C, 3000 rpm for 15 min, and then the plasma was aliquoted and stored at −80°C. The rats' livers were rinsed with normal saline, and parts of them were fixed with 4% PFA (paraformaldehyde) for histological examination. All the other liver tissues were snap-frozen in liquid nitrogen and stored at −80°C for qPCR or Western blot analysis. The epididymal white adipose tissues (EpiWAT) were also collected and weighted, and treated like liver tissues.

### 2.5. Histological Examinations of Liver Tissues and Adipose Tissues

Routine hematoxylin and eosin (H&E) staining of liver tissues and EpiWAT was carried out using 4% PFA fixing, paraffin embedding, and sectioning (4 *μ*m) as described previously [28]. Three or four sections from every tissue sample were stained. The morphology of the liver tissue and EpiWAT was observed under a light microscope of PerkinElmer Vectra 3 (PerkinElmer, USA). The cell number of the same area EpiWAT was counted and compared.

### 2.6. Biochemical Analyses

Triglyceride, total cholesterol (TC), low-density lipoprotein cholesterol (LDL-C), and high-density lipoprotein cholesterol (HDL-C) concentrations were determined using a commercial kit (Shanghai Rongsheng Biotech Co., Ltd., China). Alanine aminotransferase (ALT) and aspartate aminotransferase (AST) were detected using a commercial detection kits (Nanjing Jiancheng Bioengineering Institute, China).

### 2.7. Glucose Homeostasis

At week 19, rats were fasted overnight (12 h) and oral glucose tolerance tests (OGTT, 2 g of 50% glucose/kg body weight) were carried out as described by Wang et al. [29] with minor modification. Blood samples were collected through the tail vein, and levels of blood glucose were determined by ONETOUCH UltraEasy glucometer (Johnson, USA) before (0 min) and after glucose injection at different time points (15, 30, 60, and 120 min). The total area under the curve (AUC) was calculated for OGTT.

Blood samples were collected from the visual abdominal aorta at the end of the study. The fasting glycemia and insulinemia were determined using an ultra-sensitive ELISA kit (Alpco, USA). The homeostasis model assessment of insulin resistance index (HOMA-IR) was calculated based on the following formula: fasting insulinemia (mUI/mL) × fasting glycemia (mM)/22.5.

### 2.8. Metabolomics Analysis

Experimental nontargeted metabolomics analysis of hepatic tissues was performed as previously described [28]. Briefly, 10 *μ*L of nonadecanoic acid methanol solution (1 mg/mL, w/v), 250 *μ*L of H_2_O-MeOH-CHCl_3_ solution (2 : 5:2, v/v/v), and 50 mg of rat liver were homogenized. Then, the resulting mixture was kept at 4°C for 20 min. Next, the mixture was centrifuged at 14,000 rpm for 15 min at 4°C. Subsequently, 200 *μ*L of supernatant was dried in nitrogen. The residue was derivatized and analyzed as previously reported on 7890B-5977B GC-MS with a HP-5MS column (60 m × 0.25 mm × 0.25 *μ*m, Agilent, MA, USA).

The data analysis was performed as previously described [28]. In brief, all the GC-MS raw data were subjected to batch molecular feature extraction by using MassHunter Profinder_B.08 (Agilent Co., Ltd., CA, USA). Then, the generated data were exported to Excel (Microsoft, Redmond, WA, USA) and used in the subsequent multivariate analysis. All the raw data were stored at Guangdong Metabolic Disease Research Centre of Integrated Medicine, which will be available upon request. Unsupervised PCA (principal component analysis) and supervised OPLS-DA (orthogonal partial least-squares discriminant analysis) analysis were performed on SIMCA-P 13.0 software (Umetrics, Umeå, Sweden) to identify plasma metabolites contributing to the differences between the two groups. All variables were Pareto-scaled prior to analyses. Here, VIP (Variable Importance in Projection) >1.0 and *p* < 0.05 were set as a statistical threshold for discriminating key differential metabolites.

### 2.9. Molecular Network Construction Using IPA

Molecular network construction using Ingenuity Pathways Analysis (IPA, QIAGEN, Germany) was carried out as previously described [28, 30]. The IPA was applied to construct the metabolic interaction networks by submitting the list of modulated metabolites, the corresponding fold change, and their KEGG identity (http://www.kegg.jp), to online analysis. Based on the Ingenuity Pathway Knowledge Database, the network of interactions among metabolites, protein, and gene was generated.

### 2.10. Quantitative RT-PCR

RT-qPCR (real-time quantitative reverse transcription-polymerase chain reaction) was performed as previously reported [28, 30]. Briefly, hepatic total RNA was extracted with RNAiso plus reagent (Takara Biotechnology, Dalian, China) and was reversely transcribed to cDNA using PrimeScript™ RT kit with gDNA Eraser (Takara Biotechnology, Dalian, China). mRNA expression levels were determined using SYBR Green method on a PikoReal™ real-time PCR system (Thermo Fisher Scientific Inc., CA). Rat-specific primers for phosphatidylinositol-3 kinase (PI3K), protein kinase B (AKT), insulin receptor substrate 1 (IRS1), sterol regulatory element-binding protein-2 (SREBP-2), low-density lipoprotein receptor (LDLR), proprotein convertase subtilisin/kexin type 9 (PCSK9), and GAPDH (glyceraldehyde-3-phosphate dehydrogenase) were designed and synthesized by Sangon Biotech (Shanghai, China) (for details, see Supplementary Table S1.) The 2^(−ΔΔCT)^method was used to determine the relative expression of mRNA. The amount of each gene was normalized to the amount of rat GAPDH.

### 2.11. Western Blotting

Western blotting was conducted as previously described [28]. In brief, about 50 mg of rat's liver tissue was homogenized in 500 *μ*L RIPA (radio immunoprecipitation assay) (Solarbio Science & Tech., Beijing, China) supplemented with a PMSF (phenylmethanesulfonyl fluoride) (Solarbio Science & Tech., Beijing, China) and Protease Inhibitor Cocktail (Millipore, Calbiochem, USA). Notably, for phosphorylated protein analysis, phosphatase inhibitors (Beyotime Biotechnology, Shanghai, China) were added while the total protein was extracted. The tissue homogenates were centrifuged at 12000 rpm and 4°C for 10 min and the supernatants were collected. The total protein concentration of the tissue lysates was determined with a BCA protein assay kit (Beyotime Biotechnology, Shanghai, China). A total of 30–40 *μ*g protein from each sample was separated on SDS-PAGE (sodium dodecyl sulfate-polyacrylamide gel electrophoresis) and transferred to poly(vinylidene fluoride) (PVDF) membrane (Millipore, Darmstadt, Germany). The membrane was then blocked with 5% nonfat milk at room temperature for 1–1.5 hours, following incubation with the primary antibodies against PI3K (Rabbit polyclonal 4249S, Cell Signaling Technology, Danvers, MA, USA), AKT (Rabbit polyclonal 4685S, Cell Signaling Technology, Danvers, USA), p-AKT (Rabbit polyclonal 4060S, Cell Signaling Technology, Danvers, USA), p-PI3K (Rabbit polyclonal ab182651, Abcam, Cambridge, UK), SREBP2 (Rabbit polyclonal ab28481, Abcam, Cambridge, UK), PCSK9 (Rabbit polyclonal ab31762, Abcam, Cambridge, UK), and LDLR (Rabbit polyclonal ab30532, Abcam, Cambridge, UK) and *β*-actin (Cell Signaling Technology, Danvers, USA) at 4°C overnight. Subsequently, the membrane was incubated with appropriate HRP-conjugated secondary antibody for 1 hour and visualized by using an enhanced chemiluminescence kit (Cyanagen, Bologna, Italy). The intensity of the immunoblot signal was detected and quantified using Image Master VDS (SYNERGY Gene Company Limited, Hong Kong, China) with image analysis software (Image Master Total Lab; SYNERGY).

### 2.12. Statistical Analysis

All data are shown as means ± standard deviation (SD). Data sets that involved more than two groups were assessed by one-way ANOVA followed by Newman–Keuls post hoc tests. *p* < 0.05 was considered statistically significant. For metabolomics analysis, data were normalized to the internal standard and all variables were Pareto-scaled prior to analyses. R (https://www.r-project.org) and GraphPad Prism 6.0 software (GraphPad, CA, USA) were used for statistical analysis and graphics.

## 3. Results

### 3.1. Preparation and Quantitative Profiling of THF

**Figure 1:**
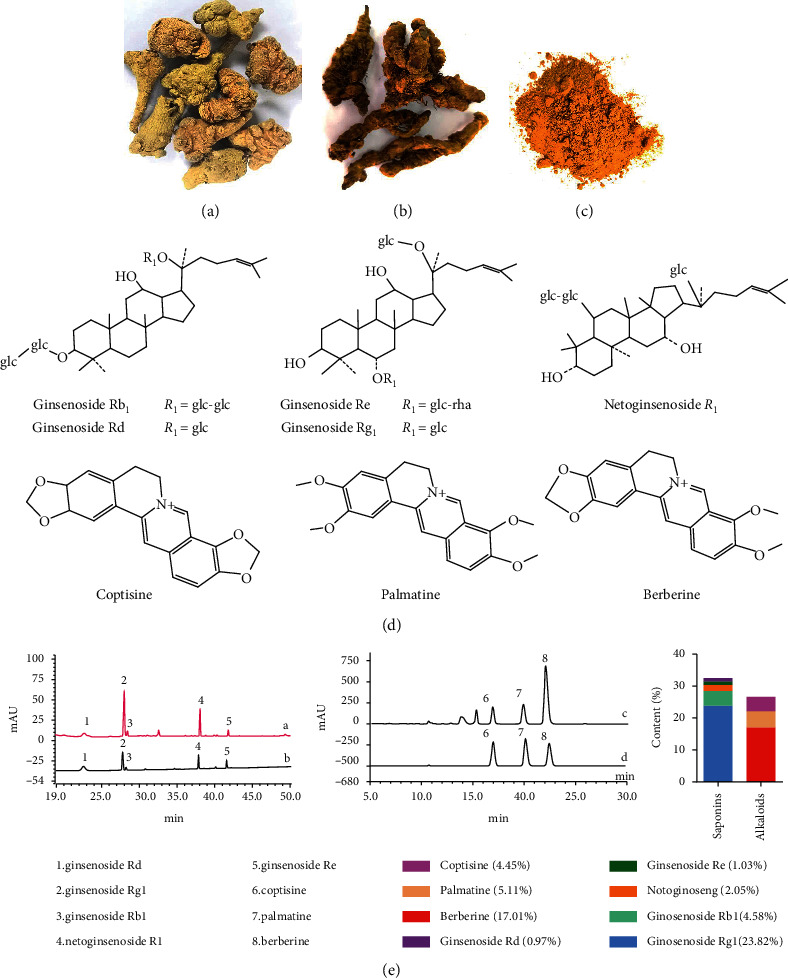
Preparation and quantitative profiling of Tian-Huang formula, a mixed extract of *Panax notoginseng* and *Coptis chinensis*. (a) *P*. *notoginseng* root and rhizome, (b) *C*. *chinensis* root, (c) dry powder of Tian-Huang formula (THF), a mixed extract of *P*. *notoginseng* and *C*. *chinensis*, (d) chemical structure of eight main components of THF, namely, ginsenoside Rb_1_, ginsenoside Rg_1_, ginsenoside Rd, ginsenoside R_1_, notoginsenoside R_1_, coptisine, palmatine, and berberine, and (e) content of abovementioned eight components analyzed by HPLC following the method recorded by Pharmacopoeia of the People's Republic of China (2015).

Saponins and alkaloids are the main bioactive ingredients of THF, and a traditional ethanol reflux extraction and macro-porous resin purification process is effective for THF preparation [20]. As shown in Figure 1, the main active components of *P*. *notoginseng* root and rhizome (Figure 1(a)) are ginsenoside Rg_1_, ginsenoside Rb_1_, ginsenoside Rd, ginsenoside Re, and *p*. *notoginseng* saponin R_1_ (Figure 1(d)). The major active components of *C*. *chinensis* root (Figure 1(b)) are berberine, coptisine, and bamatine (Figure 1(d)). The prepared dry THF is yellow brown powder (Figure 1(c)) and HPLC analysis showed that it was made of ginsenoside Rg1 (23.82%), ginsenoside Rb1 (4.58%), ginsenoside Rd (0.97%), ginsenoside Re (1.03%), *Panax notoginseng* saponin R1 (2.05%), coptisine (4.45%), bamatine (5.11%), berberine (17.01%), and some other unidentified components (41.98%) (Figure 1(e)).

### 3.2. THF Improved Lipid Accumulation in DIO Rats

**Figure 2:**
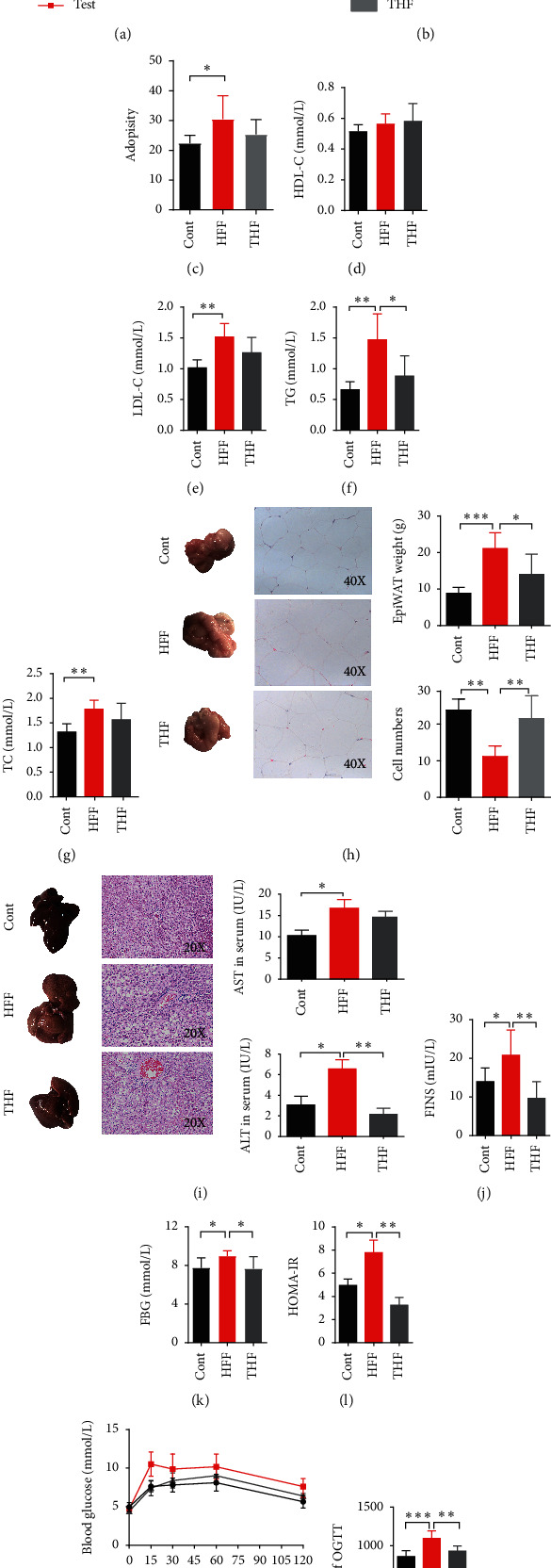
THF improves hepatic steatosis and glucose intolerance of diet induced obese rats. (a) The high-fat high-fructose diet-fed group (test; *n* = 16) and control group (cont; *n* = 8) rats were measured for body weight. (b) At the beginning of week 15, the test group rats were divided into HFF-fed and THF (200 mg/kg) treated group (THF; *n* = 8)) and HFF-fed and vehicle-treated group (HFF; *n* = 8). The control group, the HFF group, and the THF group rats were also measured for body weight from week 15 to week 19 and (c) adiposity at week 19. Plasma (d) HDL-C, (e) LDL-C, (f) TC, and (g) TG levels were also measured. (h) Representative images of EpiWAT and EpiWAT sections with H&E staining; the EpiWAT weight and cells number were measured; (i) representative images of liver and hepatic tissue sections with H&E staining; serum ALT and AST were also measured; (j) FINS and (k) FBG were measured and (l) HOMA-IR index was calculated; (m) oral glucose tolerance tests (OGTT) were carried out, and (n) OGTT-AUC was also calculated. Data are presented as mean ± SD (*n* = 6–8); ^*∗*^*p* < 0.05; ^*∗∗*^*p* < 0.01; ^*∗∗∗*^*p* < 0.001. HFF, high-fat high-fructose diet; THF, Tian-Huang formula, a mixed extract of *P*. *notoginseng* and *C*. *chinensis*; TC, total cholesterol; TG, triglycerides; LDL-C, low-density lipoprotein cholesterol; HDL-C, high-density lipoprotein cholesterol; EpiWAT, epididymal white adipose tissue; ALT, alanine aminotransferase; AST, aspartate aminotransferase; FINS, fasting serum insulin; FBG, fasting blood glucose; HOMA-IR, insulin resistance index.

HFF diet provides excessive energy and results in obesity. In order to evaluate the protective effects of THF, rats undergoing HFF diet were treated with THF (200 mg/kg) or vehicle. As shown in Figure 2(a), after 7 weeks of HFF-feeding, a significant increase in body weight was observed for the test group compared to control group rats, and the difference became more significant over time (*p*=0.002, week 14). At the end of week 14, the rats of HFF-fed test group were divided into the HFF-fed group and vehicle-treated group and the HFF-fed and THF-treated group, as described in the methods section. As shown in Figures 2(b) and 2(c), THF treatment lowered the body weight significantly (*p*=0.01); an obvious tendency towards amelioration of the rats' adiposity was also observed (but was not statistically significant). At the end of the experiment, the rats' plasma levels of TC, TG, LDL-C, and HDL-C showed an obvious rise in the HFF group (*p*=0.0018, 0.0005, 0.0005, and 0.0011, respectively; Figures 2(d)–2(g)). However, the THF treatment significantly decreased the level of rats' plasma TG (*p*=0.031) and showed a trend towards decreased levels of TC, LDL-C, and HDL-C although this was not statistically significant (Figures 2(d)–2(g)). Thus, effects of THF were predominantly noted on improving triglyceride metabolism.

Pathological changes of white adipose tissue are part of the typical characteristics of the DIO rats' model [31]. In our results, the EpiWAT weight of the HFF group was elevated significantly compared to that of the control group (*p*=0.0011) while the THF group showed a significant decrease (*p*=0.0403; Figure 2(h)). The EpiWAT tissues examined by H&E staining displayed more hypertrophic adipose cells in the HFF group than the control and THF groups; there existed a statistically significant change of the cell number of the same view EpiWAT area among the three groups (*p* < 0.01; Figure 2(h)). Thus, it was also notable that THF could attenuate the hypertrophy of adipose tissue. In addition, the accumulation of fat in the liver is a noted feature in DIO rats [31]. The phenotypes of the whole liver showed that, compared with the normal group, the liver surface color of the HFF group was paler, and the THF group was more normal than the HFF groups (Figure 2(i)). Meanwhile, the results of histological analyses of the liver tissues pathological section stained by H&E showed that there were more lipid droplets and vacuoles in the liver cells of the HFF group, which were larger and accompanied by inflammatory cell infiltration (Figure 2(i)). However, the number of lipid droplets and vacuoles in the THF group was visibly reduced. Furthermore, the serum concentrations of ALT and AST in the HFF group were significantly increased (*p*=0.0156 and 0.0183, respectively), indicating that the liver cells were damaged. After THF intervention, the ALT level was decreased significantly (*p*=0.0019), and AST was also reduced, although it was not statistically significant (Figure 2(i)). Collectively, this data demonstrated that a long period of HFF diet resulted in fat accumulation in DIO rats, and THF (200 mg/kg) treatment could provide an effective reduction.

### 3.3. THF Improved Glucose Intolerance in DIO Rats

Glucose intolerance always accompanies obesity [1]. In order to investigate whether THF can also improve the glucose metabolism disturbance of DIO rats, homeostasis tests were performed. As expected, the glucose homeostasis tests (Figures 2(j)–2(n)) showed that HFF feeding resulted in glucose intolerance, with altered FINS (fasting serum insulin), FBG (fasting blood glucose), and HOMA-IR. The tests revealed that the HFF group displayed a significant elevation of FINS compared with the control group (Figure 2(j)); similar elevations in FBG (Figure 2(k)) and HOMA-IR (Figure 2(l)) were also noted (*p*=0.0330, 0.0312, and 0.0355, respectively). Moreover, the results of the OGTTs indicated that there existed an insulin resistance and impaired glucose tolerance in the HFF group rats (Figures 2(m) and 2(n)). However, THF treatment significantly attenuated these conditions; four weeks of THF (200 mg/kg) treatment significantly improved both impaired glucose tolerance and insulin resistance (*p*=0.0066 and 0.0053, respectively) (Figures 2(j)–2(n)).

### 3.4. Metabolomics Analysis Revealed Distinct Metabolite Composition in response to THF Treatment

**Figure 3:**
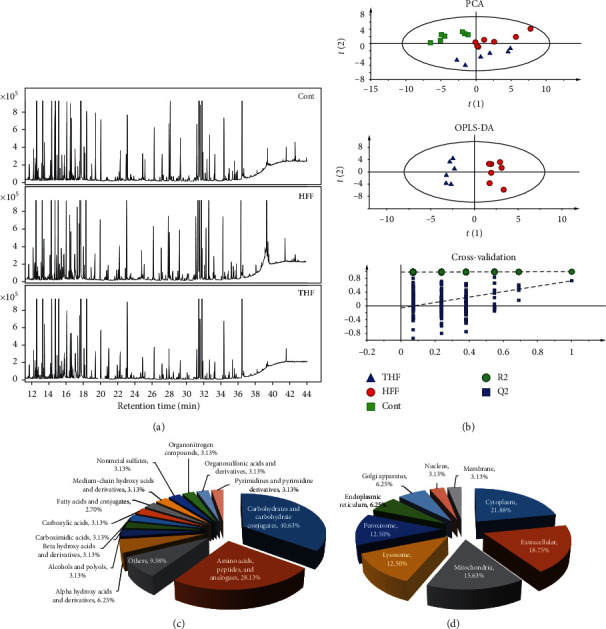
Nontargeted metabolomics analysis presented key differential metabolites between HHF-fed-THF-treated and HFF-fed-vehicle-treated rats. (a) Representative GC-MS total ion chromatograph of plasma from HFF-fed and THF (200 mg/kg) treated group (THF), HFF-fed and vehicle-treated group (HFF), and the control group (Cont) rats. (b) Multivariate statistical analysis of GC-MS metabolic profiling data. PCA scores plot and OPLS-DA scores plot were derived from GC-MS spectra of three groups of rats (THF, blue triangles; HFF, red dot; Cont, green diamond) and statistical validation of the OPLS-DA model by permutation testing. (c) Chemical classification of the key differential metabolites based on the annotations of Human Metabolome Database (http://www.hmdb.ca) and their corresponding percentage. (d) Cellular locations of the key differential metabolites based on the annotations of Human Metabolome Database (http://www.hmdb.ca) and their corresponding percentage. HFF, high-fat and high-fructose diets; THF, a mixed extract of *P*. *notoginseng* and *C*. *chinensis*; PCA, principle component analysis; PLS-DA, partial least squares-discriminate analysis; OPLS-DA, pairwise orthogonal projections to latent structures discriminate analysis.

Collectively, the above-mentioned data provide compelling evidence for a protective effect of THF on the glucose intolerance and hepatic steatosis in experimental DIO rats. However, the molecular mechanisms triggered by THF were still unclear. In order to investigate the underlying mechanisms, we conducted nontargeted metabolic profiling of the liver tissues using gas chromatography mass spectrometry (GC-MS, typical total ion chromatograms are shown in Figure 3(a)). Referring to the NIST14.0 database, a total of 215 compounds were detected and quantified by normalizing to the internal standard for all the samples. We used PCA for multivariate analysis of the nontargeted quantitative metabolic profiling data of the control, HFF, and THF (200 mg/kg) groups. The PCA scores plot displayed clear differences between the three groups (*R*^2^*X* = 0.867, *Q*^2^ = 0.501) (Figure 3(b)). In addition, OPLS-DA analysis was used to maximize the discrimination between the HFF groups and THF groups. The results revealed a clear separation between the two groups (*R*^2^*X* = 0.838, *R*^2^*Y* = 0.956, *Q*^2^ = 0.901); the values of these parameters approached 1.0, indicating a stable model with predictive reliability (Figure 3(b)). Permutation testing demonstrated that the OPLS-DA model was robust (*R*^2^ = 0.604, *Q*^2^ = −0.957) (Figure 3(b)). Based on a threshold of VIP values >1 and *p* values <0.05, 32 endogenous molecules, including oxalic acid, lactic acid, 4-hydroxybutanoic acid, palmitic acid, and stearic acid, were found to be key differential metabolites between the HFF groups and the THF groups (for details, see Supplementary Table S2). Using the HMDB (http://www.hmdb.ca) for classification of the 32 metabolites, over 40% were sub-clustered as carbohydrates and carbohydrate conjugates, about 28.13% were amino acids, peptides, and analogs; about 6.25% were alpha-hydroxy acids and derivatives (Figure 3(c)). In terms of cellular locations, these metabolites are primarily located in cytoplasm (21.88%), extracellular (18.75%), mitochondria (15.63%), lysosome (12.50%), peroxisome (12.50), endoplasmic reticulum (6.25%), and Golgi apparatus (6.25%) (Figure 3(d)). Collectively, this series of compounds allowed us to define a metabolomics signature of THF biological actions.

### 3.5. Pathway Enrichment and Ingenuity Pathway Analysis Hinted AKT and LDLR Involvement in THF Improvement of Glucose and Lipid Metabolism Homeostasis

**Figure 4:**
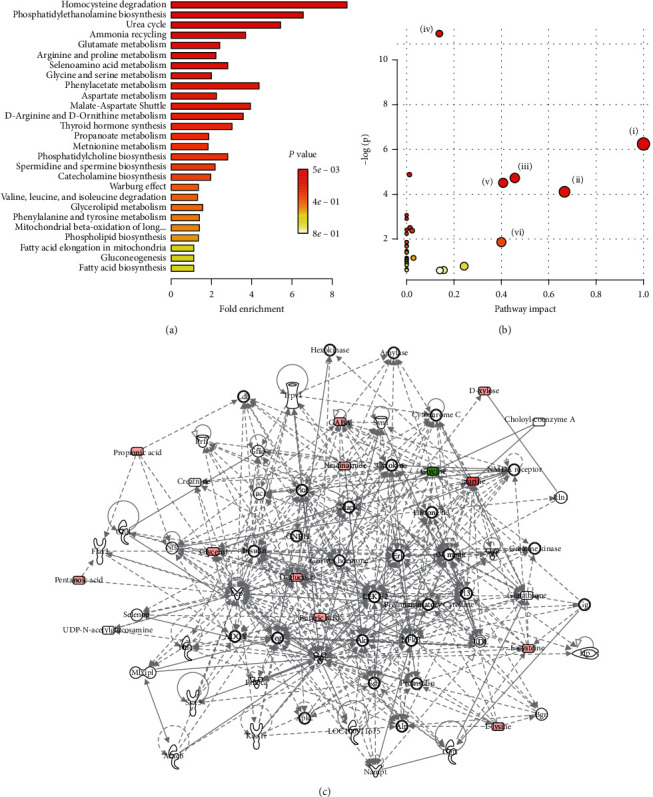
Ingenuity pathways analysis suggested that THF improves the glucose intolerance and hepatosteatosis by regulating PI3K and LDL-R involved pathways. (a) Enrichment analysis performed using the pathway-associated metabolites sets with MetaboAnalyst 4.0. (b). Overview of pathway analysis using Fisher's Exact Test as algorithms with MetaboAnalyst 4.0; (i) phenylalanine, tyrosine, and tryptophan biosynthesis, (ii) valine, leucine, and isoleucine, (iii) alanine, aspartate, and glutamate metabolism, (iv) aminoacyl-tRNA biosynthesis, (v) phenylalanine metabolism, (vi) methane metabolism. (c) Top regulated metabolic network generated by IPA software (Qiagen, Germany) based on the different metabolites discovered through metabolomics analysis hinted that THF improves the glucose intolerance and hepatic steatosis by regulating PI3K and LDL involved pathways. PI3K, Phosphatidylinositol-3 kinase; LDL-R, low-density lipoprotein receptor. HFF, high-fat and high-fructose diets; THF, Tian-Huang formula, a mixed extract of *P*. *notoginseng* and *C*. *chinensis*.

The algorithmically constructed metabolic networks can generate much more insight than discussing the key metabolites individually. In order to further address the underlying mechanisms of THF-mediated protection on DIO rats, we used specialized software to interpret and visualize the biological changes, altered canonical pathways, and metabolic networks. With the above-mentioned 32 key differential metabolites regulated by THF treatment, pathway enrichment analysis and molecular interaction networks construction were carried out using MetaboAnalyst 4.0 (http://www.metaboanalyst.ca) (Figures 4(a) and 4(b)) and Ingenuity Pathway Analysis software (http://www.qiagen.com), respectively [30, 32]. The highest-scoring (IPA score, 42) network targeted by THF involved 12 molecules, in which phosphatidylinositol-3 kinase- (PI3K-) protein kinase B (AKT), mitogen-activated protein kinases- (MAPK-) extracellular regulating kinase (ERK), extracellular regulating kinase 1/2 (ERK 1/2), insulin, proinsulin, pro-inflammatory cytokine, and LDL were algorithmically connected items (Figure 4(c)). Obviously, this network involved regulators that helped THF modulation of the glucose metabolism pathway and lipid metabolism pathway. This hinted that the hypoglycemic and hypolipidemic mechanism of THF might target PI3K and LDL-R dependent pathways, as well as pro-inflammatory related pathways.

### 3.6. qPCR and Western Blotting Results Supported the Hypothesis That THF Improved Glucose Intolerance and Hepatosteatosis by Potentially Targeting the AKT-SREBP Nexus

**Figure 5:**
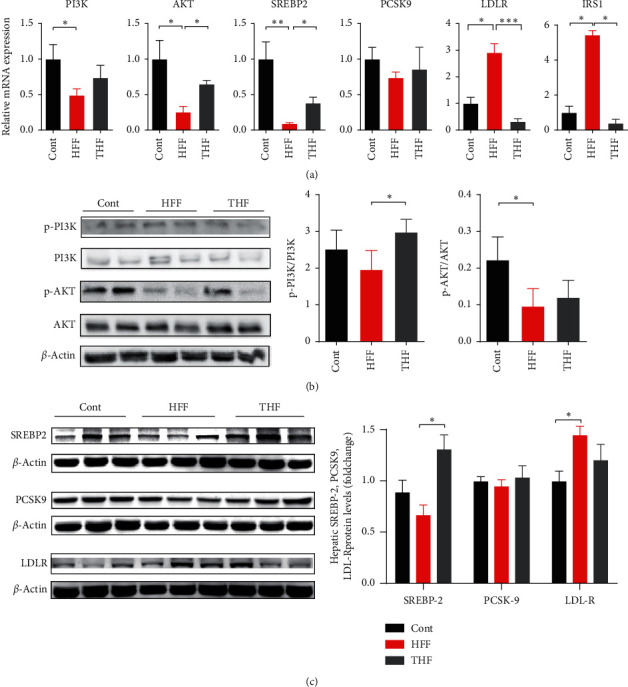
THF improves glucose intolerance and hepatosteatosis in HFF-diet induced obese rats partly through AKT-SREBP nexus. (a) qPCR results showed that the mRNA expressions of PI3K, AKT SREBP2, PCSK9, LDL-R, and IRS1 were improved after treating with THF in DIO rats, indicating that glucose and lipids metabolism pathways were maintained (*n* = 6, ^*∗*^*p* < 0.05 vs. con group; ^#^*p* < 0.05 vs HFF group). (b) Representative Western blots were shown while *β*-actin levels were used as a loading control. Western blot results showed the phosphorylation of PI3K and AKT declined in HFF group, indicating that PI3K-AKT pathway was suppressed. THF treatment attenuated the phosphorylation of hepatic PI3K and AKT in DIO rats (*n* = 4, ^*∗*^*p* < 0.05 vs. con group; ^#^*p* < 0.05 vs HFF group). (c) Representative Western blots were shown while *β*-actin levels were used as a loading control. Densitometric analysis results showed that HFF decreased the protein levels of SREBP-2 and increased the protein levels of LDL-R, which was reversed by THF treatment (*n* = 6, ^*∗*^*p* < 0.05 vs. con group; ^#^*p* < 0.05 vs HFF group). PI3K, phosphatidylinositol-3 kinase; AKT, protein kinase B; IRS1, insulin receptor substrate 1; SREBP-2, sterol regulatory element-binding protein-2; PCSK9, proprotein convertase subtilisin/kexin type 9; LDL-R, low-density lipoprotein receptor.

Since PI3K-AKT is the canonical pathway of energy metabolism, and SREBP2-PCSK9-LDL-R pathway is reported to play a vital role in hepatic lipids metabolism, and combined with the aforementioned clues from IPA generated molecular networks, we hypothesized that THF improved glucose intolerance and hepatosteatosis in DIO rats by potentially targeting the AKT-SREBP nexus [33]. The mRNA expression and the protein levels of two canonical pathways, namely, PI3K-AKT and SREBP2-PCSK9-LDLR, were thus analyzed using RT-qPCR and Western blotting. As expected, the relative mRNA expressions of PI3K, AKT, and SREBP2 were significantly downregulated in HFF group compared with control group (*p*=0.0066, 0.0097, and 0.0086, respectively), while they were upregulated after THF treatment compared with the HFF group (Figure 5(a)). PCSK9 had the same tendency although it was not statistically significant. In contrast, the relative mRNA expressions of LDLR and insulin receptor substrate 1(IRS1) were upregulated in the HFF group while both of them were downregulated in the THF group (Figure 5(a)). Furthermore, confirming our hypothesis, THF could restore the phosphorylation of PI3K and AKT to homeostatic levels, which were blocked by HFF feeding, as evaluated by Western blotting (Figure 5(b)). Likewise, the protein levels of hepatic SREBP2, PCSK9, and LDLR were also measured. As shown in Figure 5(c), whilst HFF feeding inhibited SREBP2 protein levels, THF treatment resulted in a significant increase (*p*=0.0027), suggesting that THF supported SREBP2 activity to maintain the homeostatic cascade. Moreover, compared with control group, the HFF diet induced a marked increase in the protein level of LDLR (*p*=0.0135), which was attenuated by THF treatment (Figure 5(c)). However, as for the protein level of hepatic PCSK9, there were no fluctuations, and this was in line with data on mRNA expression (Figures 5(a) and 5(c)). Based on these results, we concluded that THF reversed the metabolic disturbance by targeting AKT-SREBP nexus.

## 4. Discussion

In the present study, we used a simple but robust process to prepare THF following the Ch.P. instructions and animal test demonstrated that THF could improve metabolic disturbance through the AKT-SREBP nexus. Our results proved that THF has valuable therapeutic potential for human DIO and its complications.

We chose the HFF diet induced rat model to evaluate the improvement effect of THF on glycolipid metabolism, because it is a well-accepted model of DIO and its complications, such as dyslipidemia, impaired glucose tolerance, and insulin resistance, and even its more serious consequences, T2DM, NAFLD, and metabolic syndrome [34, 35]. In fact, HFF simulated perfectly the popular Western diet and reflected the dietary preference of a large number of people [34], which can lead to obesity and NAFLD [36].

THF is a traditional Chinese medicinal formula composed of *P*. *notoginseng* and *C*. *chinensis*. Given the long traditional medicine history of *P*. *notoginseng* and *C*. *chinensis*, quite a few of studies have tried to reveal their pharmacological functions and mechanisms from different standpoints. Guo et al. used PNS to treat diabetes and found its molecular mechanism related to reducing skeletal muscle insulin resistance by regulating the IRS1-PI3K-AKT signaling pathway and GLUT4 expression [37]. Ding et al. investigated the hepatoprotective effects of PNS and found it can improve liver lipid accumulation and oxidative stress and protect acute ethanol-induced liver injury [15]. Zhong et al. also claimed that PNS can promote liver regeneration by activating PI3K/AKT/mTOR cell proliferation pathway and upregulating AKT/Bad cell survival pathway [38]. As for CCA, there are also some studies showing their benefits for therapeutic intervention on obesity and its complications. For example, Choi et al. [39] reported that CCA exerted anti-adipogenic activity on 3T3-L1 adipocytes 2 by downregulating C/EBP-*α* and PPAR-*γ*; Yang et al. [40] revealed that CCA may help alleviate hyperglycemia in diabetes by promoting glucose uptake by skeletal muscles; and Li et al. [41] concluded that CCA prevent diabetic cognitive deficits most likely by ameliorating disorder of glucose and lipid metabolism, attenuating A-*β* deposition, and enhancing insulin signaling. Without exception, in this study, we found that THF can significantly improve liver lipid accumulation and impaired glucose tolerance, and reduce fasting blood glucose. It is notable that, during our previous research, the effects of different doses of THF on lipid-lowering were compared and 200 mg/kg dose (THF weight to rat body weight) had the best effect [20–23]. Herein, in the present mechanism investigation, 200 mg/kg of THF treatment was performed.

The endogenous metabolite spectrum reflects phenotypic changes, and accurate regulation of levels of all kinds of endogenous molecules is critical for metabolic homeostasis. Viewed as the list of key metabolites derived from our metabolomics analysis, a HFF diet contributes to marked changes of a series of endogenous molecules, such as 4-hydroxybutanoic acid, oxalic acid, lactic acid and taurine, etc. These changes must be the result of a series of physiological and biochemical reactions and there likely exists some kind of underlying linkage. Using software algorithm-based enrichment and IPA analysis, the full list of modulated key metabolites presented us a molecular network revealing the delicate and intricate influence of THF on rats' metabolism. Along with our knowledge on signaling pathways, we hypothesized that the AKT-SREBP nexus could play a vital role mediated THF's protection, of which the PI3K-AKT pathway and SREBP2-PCSK9-LDLR pathway are familiar pathways related to energy and lipid metabolism [33]. Indeed, some saponin components of the THF described here, such as ginsenoside Rb1 and ginsenoside Rg1, have been reported to target PI3K-AKT pathway to regulate innate immune responses in macrophages [42], to protect IL-1*β*-induced mitochondria-activated apoptosis [43], to mitigate oxidative stress and apoptosis [44], and to prevent homocysteine-induced endothelial dysfunction [45]. Similarly, as for the other three alkaloid components of THF, namely, berberine, coptisine, and palmatine, literature demonstrates that they can reduce ischemia/reperfusion-induced myocardial apoptosis in diabetic rats [46], can ameliorate insulin resistance in obese rats [47], and can inhibit IL-21/IL-21R mediated inflammatory proliferation [48], through the PI3K-AKT pathway. Moreover, berberine has been claimed to attenuate nonalcoholic hepatic steatosis through the AMPK-SREBP-1c-SCD1 pathway [49], alleviate adipogenesis via the AMPK-SREBP pathway [50], and increase the expression of PCSK9 levels in HFD rats through the SREBP-2 pathway [51]. In line with these, our data show that THF has a strong potential to regulate the AKT-SREBP nexus specifically to improve metabolic disorders upon high-fat high-fructose diet challenge. However, we found that neither the mRNA nor the protein expression levels of PCSK9 were altered, and both the mRNA and the protein expression level of LDLR showed inverse changes. These findings are not completely consistent with the aforementioned literature, which suggests that THF may have different and complicated effects compared to single compounds, possibly affecting some other mediators not focused on in this study but belonging to the AKT-SREBP nexus. Recent studies have shown that berberine significantly upregulated the mRNA level of SREBP-2 in the liver of HFD rats, thereby increasing the levels of PCSK9 and LDLR [51]. In this study, THF increased the expression of SREBP2 and PCSK9 and decreased the expression of LDLR, which was linked with the protein levels assessed by Western blotting analysis. Thus, our study confirmed previous studies and provided novel additional important information regarding the effects of THF on glucose intolerance and lipid accumulation.

**Figure 6:**
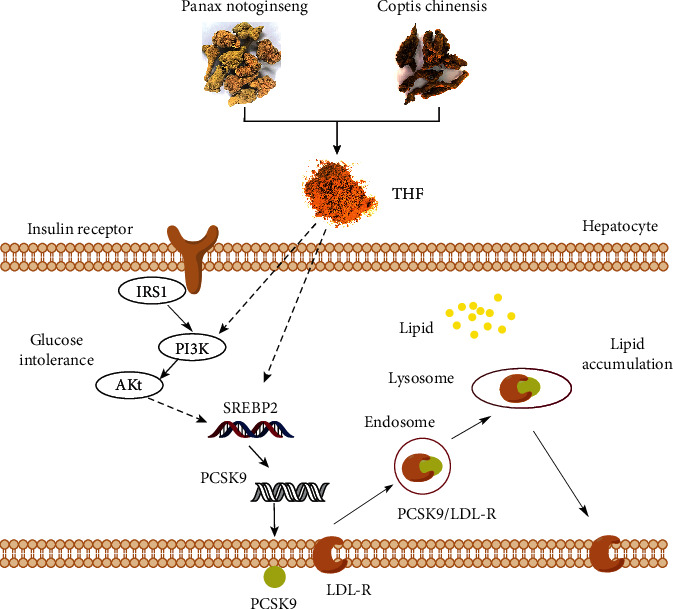
Schematic model summarizing the proposed underlying mechanisms of the THF ameliorates glucose intolerance and hepatosteatosis by maintaining the homeostasis of AKT- SREBP nexus in DIO rats. HFF, high-fat and high-fructose diets; THF, Tian-Huang formula, a mixed extract of *P*. *notoginseng* and *C*. *chinensis*; IRS1, insulin receptor substrate 1; PI3K, phosphatidylinositol-3 kinase; AKT, protein kinase B; SREBP-2, sterol regulatory element-binding protein-2; PCSK9, proprotein convertase subtilisin/kexin type 9; LDL-R, low-density lipoprotein receptor.

Thus, although a full understanding of the mechanisms underpinning THF-mediated protection in DIO associated glucose intolerance and hepatosteatosis requires further work, we propose that THF targets the AKT-SREBP nexus to maintain glycolipid metabolic homeostasis. By targeting the cascades, THF may protect against DIO associated glycolipid metabolic disturbance in a subtle and effective way (Figure 6). Therefore, our study indicates that there is great medicinal potential to be found in THF. Meanwhile, in our future research, the therapeutic potential of the isolated compounds or fractions from THF should be evaluated. Furthermore, THF has a long history of usage in Chinese medicine, and it is currently employed in clinic, suggesting that THF might not have significant drawbacks or side effects often associated with other hypoglycemic and hypolipidemic therapies. Thus, the chemical material basis elucidated here might provide more evidence for the rational development of novel drugs to treat human DIO accompanied impaired glucose metabolism and hepatic fat accumulation conditions, even potentially T2DM and NAFLD.

## 5. Conclusions

In conclusion, this study showed that long-term HFF diet feeding can induce DIO associated glucose intolerance and hepatic steatosis in rats and that THF (200 mg/kg) treatment can ameliorate these conditions. Metabolomics analysis and algorithmically constructed metabolic networks hinted that AKT and LDLR are involved in THF's pharmaceutical effects, and qPCR and Western blotting results supported the hypothesis that THF improves glucose intolerance and hepatosteatosis in DIO rats by potentially targeting AKT-SREBP nexus. Although translation of the finding of rodent experiments to human is not so satisfied, the study sheds light on the therapeutic potential of THF against impaired glucose and lipid accumulation.

## Data Availability

All the raw data are stored at Guangdong Metabolic Disease Research Centre of Integrated Chinese and Western Medicine, which will be available from the corresponding author on reasonable request.

## Additional Points

High-fat high-fructose diet has deleterious consequences on glucose and lipid metabolism. Tian-Huang formula (THF) is composed of *P*. *notoginseng* and *C*. *chinensis*. THF alleviated hepatosteatosis and glucose intolerance in diet induced obese rats. AKT-SREBP nexus are potential targets of THF.

## Supplementary Materials

Supplementary Materials Supplemental Table S1: the key differential metabolites between HHF-fed-THF-treated and HFF-fed-vehicle-treated rats presented by metabolomics analysis. Supplemental Table S2: primer sequences for RT-qPCR. Supplemental methods: quantitative profiling of THF, a mixed extract of *Panax notoginseng* and *Coptis chinensis*. 1.1: quantitative profiling of *Panax notoginseng* saponins. 1.2: quantitative profiling of *Coptis chinensis* alkaloids. 1.3: linear regression was performed.
