# Supplementary material for: Tian-Huang Formula, a Traditional Chinese Medicinal Prescription, Improves Hepatosteatosis and Glucose Intolerance Targeting AKT-SREBP Nexus in Diet-Induced Obese Rats
Source: Evid Based Complement Alternat Med. 2021 Mar 6;2021:6617586. doi: 10.1155/2021/6617586 (PMC7955866; doi:10.1155/2021/6617586)
Supplement: Supplementary Materials — Supplemental Table S1: the key differential metabolites between HHF-fed-THF-treated and HFF-fed-vehicle-treated rats presented by metabolomics analysis. Supplemental Table S2: primer sequences for RT-qPCR. Supplemental methods: quantitative profiling of THF, a mixed extract of Panax notoginseng and Coptis chinensis. 1.1: quantitative profiling of Panax notoginseng saponins. 1.2: quantitative profiling of Coptis chinensis alkaloids. 1.3: linear regression was performed. [file 6617586.f1.pdf]

Supplemental materials:

Supplemental Table S1 The key differential metabolites between HHF-fed-THF-treated and HFF-fed-vehicle-treated rats presented by metabolomics analysis.

NO	Metabolites	VIP values [a]	*p*-values	Fold Change [b]	KEGG ID
**1**	4-aminobutanoic acid	1.61	0.02	0.45	C00334
**2**	Amphetamine	2.08	0.00	0.34	C07514
**3**	Oxalic acid	1.58	0.02	0.26	C00209
**4**	ethylene glycol	1.46	0.05	-1.85	C01380
**5**	Diethanolamine	1.49	0.03	-0.97	C06772
**6**	cyclopentanol	1.92	0.00	-0.33	C02020
**7**	cyclohexanol	1.93	0.01	-0.93	C00854
**8**	Phenylalanine	1.58	0.01	-1.11	C02057
**9**	2-oxobutanoic acid	1.49	0.02	-0.53	C00109
**10**	DL-Ornithine	1.48	0.02	0.62	C07997
**11**	L-Serine	1.44	0.03	-1.02	C00077
**12**	Ethanolamine	1.46	0.02	1.05	C00189
**13**	D-Lyxose	1.57	0.03	0.63	C00476
**14**	D-Erythrose	1.50	0.02	-1.61	C01796
**15**	L-Tyrosine	1.52	0.01	-0.59	C00082
**16**	Palmitic Acid	1.84	0.00	-0.50	C00249
**17**	Pentadecylic acid	1.57	0.03	1.17	C16537
**18**	Stearic acid	1.85	0.01	-0.53	C01530
**19**	L-Tryptophan	1.65	0.01	-0.52	C00078
**20**	Cadaverine	1.76	0.01	-1.68	C01672
**21**	D-Xylose	1.90	0.00	1.36	C00181
**22**	Batyl alcohol	2.17	0.01	-0.75	C13858
**23**	D-Threose	1.68	0.01	-0.98	C06463
**24**	4-Hydroxybutanoic acid	1.96	0.05	-1.08	C00989
**25**	Urea	1.58	0.02	0.39	C00086
**26**	Lactic Acid	1.09	0.02	-0.75	C00186
**27**	L-Glutamine	1.77	0.02	-0.98	C00064
**28**	Pyroglutamic acid	1.73	0.02	-0.68	C01879
**29**	L-Aspartic acid	1.84	0.03	1.12	C00049
**30**	L-Valine	2.11	0.03	-0.55	C00183
**31**	L-Isoleucine	1.42	0.04	0.44	C00407
**32**	L-Serine	1.52	0.03	-0.69	C00065

[a] Based on OPLS-DA results, the metabolites with variable influence on projection (VIP) values >1.0 and *p*-values < 0.05 were deemed as differential metabolites.
[b] Fold change was calculated as the logarithm of the average mass response (area) ratio between the two groups (i.e., Fold change = $\log_2$(THF /HFF).

Supplemental Table S2 **Primer sequences for RT-qPCR**

Primers	Forward primer	Reverse primer	Size(bp)
SREBP-2	GATGATGCCGACTCTACTGCTGTG	AAGGTGACCGAGGAGCGTGAG	120
LDLR	CAAGGTGACATGGCTGGCAGAG	ATCTCGTCCTCCGTGGTCTTCTG	194
PCSK9	CAGGACGAGGACGGAGACTACG	CTTGGAGCAACGGCGGAAGG	114
PI3K	CTGTGCCTTCTGCCTTACGGTTG	GCAATCGTCGTGGCGTCCTTC	83
AKT1	GGCAGGAGGAGGAGACGATGG	TTCATGGTCACACGGTGCTTGG	109
IRS1	AGCAACAGCAGCAGCAGTCTTC	ACTCTTCCGAGCCAGTCTCTTCTC	189

Supplemental methods

Quantitative profiling of THF, a mixed extract of *Panax notoginseng* and *Coptis chinensis*

1.1 Quantitative profiling of *Panax notoginseng* saponins

The temperature of the column was set at 25 ℃, and the flow rate was 1.0 ml/min. The injection volume was 10 μL. The detection wavelength was 203 nm, and the acetonitrile (A) and water (B) were used as the mobile phase. The gradient program for the ginsenoside samples was optimized as follows: 0-20 min, 20% A; 20-45 min, 20-46% A; 45-55 min, 46-55% A.

1.2 Quantitative profiling of *Coptis chinensis* alkaloids

The temperature of the columns was set at 30 ℃, and the flow rate was 1.0 ml/min. The injection volume was 10 μL. The detection wavelength was 345 nm, and acetonitrile-0.05mol/L KH_2PO_4 solution was used as the mobile phase (0.31g of sodium dodecyl sulfate was added per 100mL, and the pH value was adjusted by phosphoric acid to 3.5). The elution procedure was designed to isocratic elution (0-60 min).

1.3 Linear regression was performed

by taking the sample quantity as the horizontal coordinate (X) and the peak area as the vertical coordinate (Y). The regression equations of ginsenoside Rg1, ginsenoside Rb1, ginsenoside Rd, ginse noside Re, panax notoginseng saponin R1, coptidine, bamatine and berberine were obtained successi vely: Y_1=18.6022X1+0.0680 (r=0.9995)，Y_2=36.8398X2+0.0157(r=0.9994)，Y_3=43.8432X3+0.00 84(r=0.9995)，Y_4=42.1264X4+0.0192(r=0.9987)，Y_5=52.9536+0.0680(r=0.9993)，Y_6=572.9329 X6-10.2098(r=0.9999)，Y_7=657.7388X7-11.6213(r=0.9999)，Y_8=598.3541X8-11.2877 (r=0.9998). The content of mixed extract was determined by external standard method.
